# Abstract and Proportional Myoelectric Control for Multi-Fingered Hand Prostheses

**DOI:** 10.1007/s10439-013-0876-5

**Published:** 2013-08-09

**Authors:** Tobias Pistohl, Christian Cipriani, Andrew Jackson, Kianoush Nazarpour

**Affiliations:** 1 Institute of Neuroscience, Newcastle University, Henry Wellcome Building, Framlington Place, Newcastle upon Tyne, NE2 4HH UK; 2 The BioRobotics Institute, Scoula Superiore Sant’Anna, Viale Rinaldo Piaggio 34, 56025 Pontedera, PI Italy

**Keywords:** Electromyography, Robotic Hands, Prosthetic Control, Virtual Control

## Abstract

Powered hand prostheses with many degrees of freedom are moving from research into the market for prosthetics. In order to make use of the prostheses’ full functionality, it is essential to study efficient ways of high dimensional myoelectric control. Human subjects can rapidly learn to employ electromyographic (EMG) activity of several hand and arm muscles to control the position of a cursor on a computer screen, even if the muscle-cursor map contradicts directions in which the muscles would act naturally. But can a similar control scheme be translated into real-time operation of a dexterous robotic hand? We found that despite different degrees of freedom in the effector output, the learning process for controlling a robotic hand was surprisingly similar to that for a virtual two-dimensional cursor. Control signals were derived from the EMG in two different ways, with a linear and a Bayesian filter, to test how stable user intentions could be conveyed through them. Our analysis indicates that without visual feedback, control accuracy benefits from filters that reject high EMG amplitudes. In summary, we conclude that findings on myoelectric control principles, studied in abstract, virtual tasks can be transferred to real-life prosthetic applications.

**Electronic supplementary material:**

The online version of this article (doi:10.1007/s10439-013-0876-5) contains supplementary material, which is available to authorized users.

## Introduction

Improvements in robotics have advanced the design of hand prostheses to rival the functionality of a human hand. Some designs with multiple degrees of freedom are now entering the market for patients, like the i-limb (Touch Bionics, Livingston, UK), the bebionic (RSLSteeper, Leeds, UK) or the Michelangelo hand (Ottobock, Duderstadt, Germany). However, myoelectric control of current hand prostheses cannot compete with the dexterity and versatility of a human hand. One limitation is that the measurement of reliable and sufficiently independent signals of surface electromyography (EMG) from several muscles is difficult in amputees. Therefore, current commercial implementations of hand prostheses usually employ only one or two EMG channels and an on–off control mechanism to switch between different modes of operation or grasp types.^11^

However, if the limitation of myoelectric sources can be overcome, humans are well able to use multiple muscles for myoelectric control, as has been demonstrated in healthy subjects: Radhakrishnan *et al.* 13 showed that subjects could learn to control a cursor in two dimensions on a computer screen through EMG activity recorded during isometric contractions of multiple upper-limb muscles. In their setup, the magnitude of the EMG from six sites on hand and arm proportionally controlled cursor position in one of six directions each, with the momentary position of the cursor determined by the vector sum over all six contributions. It has been shown that control of hand muscles is sufficiently flexible to form unnatural synergies appropriate for a multitude of complex abstract functions.^9^,^17^

This encourages our view that well established and accessible paradigms of two-dimensional cursor control could be used to answer relevant questions about myoelectric control of prosthetic hands. There are, however, profound differences between a simple cursor control and the operation of a multi-fingered prosthesis; for instance, mechanical constraints and dynamics of an artificial limb may influence control proficiency. Moreover, the inherent redundancy in mapping multiple EMGs to two-dimensional cursor movements will have to be sacrificed if several actuators of a prosthetic device are to be controlled independently. It may be questioned if such a direct low-level control of multiple degrees of freedom is even feasible without excessive training.

In this manuscript, we offer a proof of concept, comparing direct posture control of a state-of-the-art robotic hand with myoelectric position control of a cursor on a screen with respect to training time and accuracy. We hypothesized that, with training, the human motor cortex can internalize the new control scheme in both cases. Similar to the previously described cursor control,^9^,^13^ we used a proportional control scheme based on the magnitude of EMG from four different muscles to afford test subjects with a high level of flexibility and access to a continuum of possible hand postures.

We asked whether the method by which the magnitude of muscle contractions is extracted in real-time would have an effect on subjects’ abilities to control either kind of task. Saunders and Vijayakumar^16^ showed that uncertainty in the controller of a hand prosthesis can lead to large errors when no feedback of the prosthesis’ operation is provided. This illustrates the importance of a reliable control signal, especially when visual feedback is impaired. We therefore implemented and compared two different estimators of muscle activity: a simple linear filter that calculates the mean absolute value of the EMG and a Bayesian filter approach that models the control signal as a combined jump and diffusion process.^15^ In a separate experiment, we evaluated the efficacy of myoelectric cursor control with either method when visual feedback was withheld to test whether the choice of the estimator may influence subject learning and performance.

## Materials and Methods

### Robotic Hand

For part of our experiments, subjects interacted with the latest version of the SmartHand,^1^ a bio-inspired hand prosthesis in which five motors independently actuate thumb abduction, thumb flexion, index finger flexion, middle finger flexion and a combined flexion of ring and little finger. Four of those motors were controlled by subjects in this study, whereas thumb abduction stayed at a constant level throughout the experiment. Bidirectional communication between prosthesis and computer was established over a serial RS232 communication protocol, using high level commands, built into the hand’s controller, to repeatedly update levels of finger flexion and monitor actual finger positions.

### Muscle Activation Estimators

Subjects used muscle contractions to control movements, either of a two-dimensional cursor or a robotic hand. The control algorithm consisted of two parts: a muscle activation estimator and a mapping strategy that linked muscle activation to the effectors. This section describes two different muscle activation estimators; mapping procedures used in the respective experiments are described under “Experiment 1” and “Experiment 2” sections.

**Figure 1:**
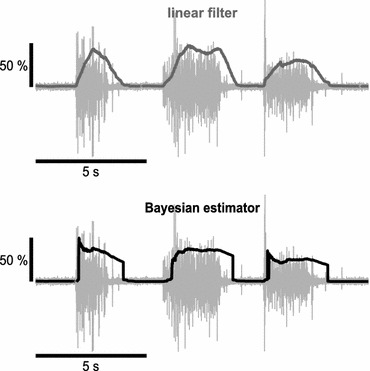
Estimators of muscle activation. Top: 15 s of EMG, recorded from 1DI muscle (light grey), overlaid with activation levels returned by the linear filter (dark grey). Bottom: same EMG, processed by the Bayesian estimator (black), dominated by a jump and a drift component. Scaling of the muscle activation levels (linear filter and Bayesian estimator) is given by the vertical bar, in % of the comfortable contraction level (assessed during calibration)

Muscle activation levels *y* were estimated online from the EMG recordings after removing any possible signal offset. We used either (1) a simple linear filter or (2) a Bayesian estimator. Theoretically, activation levels returned by either method should be equivalent during constant muscle contractions. However, these two filters exhibit very different dynamics when tracking varying EMG activity levels, as illustrated in Fig. 1.

For each channel, the linear filter averaged the rectified EMG signal of the preceding 750 ms. Although this procedure slowed the effector movement, because of continuous updating, changes in EMG started to take effect already with the next update step. The EMG was smoothed by this process, however, measurements of surface EMG often show considerable variability even during periods of constant muscle contraction, which is still reflected in the filtered signal.

The Bayesian estimator we used was a recursive filter algorithm, proposed by Sanger,^15^ updating the posterior probability density of a desired “neural drive” signal with each new sample of EMG. The rectified EMG is modeled as a random process with an exponential density; the desired neural drive as a combined diffusion and jump process. As illustrated in Fig. 1, fast onsets of the EMG are modeled more truthfully whereas, during sustained contractions, signal change is restricted to a slow drift. Following the suggestions of Sanger,^15^ we clipped the EMG at ±3 × standard deviation (as assessed during calibration) to avoid modeling the EMG density for rare extreme values. Further explanation on the calculation of control signals can be found in the Supplementary Materials.

In contrast to the smooth and continuous trajectories, generated by the linear filter, the Bayesian estimator produced sudden jumps upon rapid EMG activation or deactivation because it modeled the EMG probability density function with an exponential function to take higher order statistics of the EMG into account.^10^

### Experimental Setup (EMG Recordings, Calibration)

**Figure 2:**
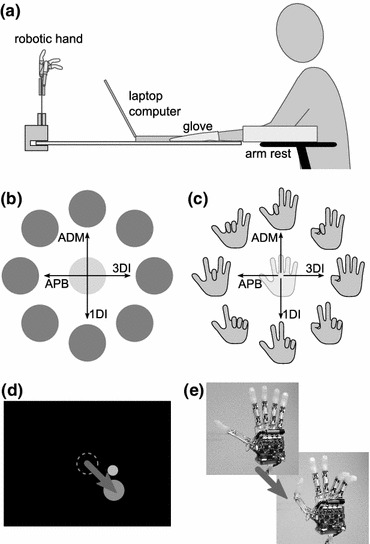
Experiment 1. (a) Subjects were facing a laptop screen and a vertically mounted robotic hand. EMG was recorded from four muscles of their left hand, which was immobilized in a horizontally fixated glove. (b) Mapping for the center-out task. Each muscle controlled movements in one DoA, the linear sum of which determined the two-dimensional position of a cursor. (c) Layout of target postures for the robotic task. The circular arrangement of hand postures illustrates parallels to the center-out mapping. However, this two-dimensional arrangement does not capture the whole space of possible hand-configurations, as each muscle directly controlled one out of four flexion levels on the robotic hand. (d) Cursor movement to targets on the computer screen. The arrow and outline of the starting position are for illustration only. (e) The starting and a sample target posture of the robotic hand

Participants sat with their left hand restrained in an open, pronated posture inside a glove, fixed to a horizontal board and their forearm strapped to an armrest (Fig. 2a). EMG was recorded from four intrinsic hand muscles of the left hand: the abductor pollicis brevis (APB, abducts the thumb in the direction of the palm), the first dorsal interosseous (1DI, abducts the index finger towards the thumb), the third dorsal interosseus (3DI, abducts the middle finger towards the ring finger) and the abductor digiti minimi (ADM, abducts the little finger away from the other fingers). Subjects controlled the myoelectric interface with isometric muscle contractions.

EMG was measured using a pair of stick-on electrodes (Bio-logic, Natus Medical Inc., Mundelein, IL, USA) positioned on the belly of the hand muscle and an adjacent knuckle. For experiment 1, an in-house fabricated (Newcastle University), battery-powered portable amplifier; for experiment 2, NeuroLog amplifiers (NL844/NL820A, Digitimer, Hertfordshire, UK) were used. In both experiments EMG amplification gains were set between 0.1 K and 5 K and signals were band-pass filtered between 30 Hz and 2 kHz. A data acquisition card (NI USB-6229, BNC, National Instruments, Austin, TX, USA) digitized the signals at a 5 kHz sampling frequency and made them available to the computer for recording and real-time processing. Data recording, online processing and graphical user-interface were handled by Python-based software, developed to implement these experiments.

For each subject, we initially recorded calibration data to assess resting levels *y*_r_ and comfortable contraction levels *y*_c_ for each EMG channel. Comfortable contractions reflected muscle activation that could easily be repeated hundreds of times. Offline verification in our earlier studies showed that comfortable contraction levels fall typically between 10% to 15% of maximum voluntary contraction.^9^,^13^ During the experiments, resulting calibration levels, *y*_r_ and *y*_c_, of EMG signals were used to normalize muscle activation levels *y*, extracted from raw EMG measurements (see “Muscle Activation Estimators”), to compute

1 $$\check{y} =(y - y_{\text{r}} )/(y_{\text{c}} - y_{\text{r}} ) $$

Mean and standard deviation were calculated from the recorded calibration data of each channel (5 s rest and 5 s comfortable contraction) to estimate measurement offset and typical signal amplitudes, respectively.

### Experiment 1

Experiment 1 was designed to test whether subjects could learn to control a prosthetic hand (experiment 1B) to a comparable accuracy and on a similar timescale as a computer cursor (experiment 1A) applying similar control schemes and whether the choice of muscle activation estimator had an effect on subject learning. Each part (A and B) consisted of two consecutive blocks that were distinguished by the type of muscle activation estimator used. Each block took about 12 min to complete and consisted of 160 trials. The order of parts A and B, as well as the order of blocks within each part, using linear or Bayesian filter, respectively, was counterbalanced (see Table 1). This design allowed that four different groups of two subjects each experienced all four experimental blocks, but each group in a different order.

**Table 1:** Order of execution in experiment 1

	Group 1	Group 2	Group 3	Group 4
Order of execution	**A**_Bayes_, **A**_Linear_,	**A**_Linear_, **A**_Bayes_,	**B**_Bayes_, **B**_Linear_,	**B**_Linear_, **B**_Bayes_,
	**B**_Bayes_, **B**_Linear_	**B**_Linear_, **B**_Bayes_	**A**_Bayes_, **A**_Linear_	**A**_Linear_, **A**_Bayes_

Four group of two subjects each completed four sequences of 160 in different order. **A**: part A (cursor control); **B**: part B (robotic hand control); subscript “Linear”: linear filter for estimation of muscle activation; subscript “Bayes”: Bayesian estimator used

We chose mapping schemes in which the magnitude of EMG, as determined by the respective muscle activation estimator, controlled position of the effector. This had several advantages: It allowed for clear predictions of necessary muscle activity to reach a certain position. Reset to a starting position was straightforward, quick and intuitive, by relaxing muscles. And despite a long filter window of 750 ms in case of the linear filter, onset of muscle activation resulted in an immediately and noticeable, albeit decelerated, effector movement.

Subjects were not told which muscle activations resulted in which actions. In order to slow down the learning process and better observe improvements over time, we designed the association between muscles and effector movements (Figs. 2b and 2c) to be non-intuitive for either task, that is, unrelated to the direction of natural hand movements (part A) or unrelated in finger selectivity (part B). Mappings were equal for all subjects.

#### Experiment 1A

For the cursor control task, subjects sat in front of a laptop computer screen and controlled the position of a circular, yellow cursor (diameter: 14.5 mm). Targets were indicated by larger, green circles (diameter: 29 mm) and located in one of eight positions on a circle of 52 mm radius. New cursor positions were calculated at an average rate of 226 Hz, well above the 60 Hz refresh rate of the screen.

Relaxing all muscles brought the cursor to the center of the screen, whereas contraction of each single muscle drove the cursor away from the center, along its direction of action (DoA, see Fig. 2). The 2D cursor position **x** was thus determined by the sum over all four DoA vectors scaled proportionally to the normalized muscle activation level $\check{y}$ of each corresponding muscle:

2 $$ {\mathbf{x}} = \mathop \sum \limits_{i = 1}^{4} \check{y}_{i}
\text{DoA}_{i} $$

This means that four independent control signals—each bounded on the lower end, since $\check{y}$ could not be negative—were mapped to two degrees of freedom of the cursor task (cf. Fig. 2b).

Targets were presented in a pseudo-random order, with each target appearing once in a set of eight consecutive trials. As illustrated in Fig. 2, four targets could be reached by activation of a single muscle at a level corresponding to 75% of comfortable contraction; the remaining four required the activation of at least two muscles simultaneously, albeit at a lower level, following vector addition in (2) (≈53% comfortable contraction). Since cursor position resulted from a sum of all muscle contributions, equal activation of two muscles with opposite DoAs could cancel out their respective effects on cursor position.

At the start of each new trial, subjects were required to relax their muscles in order to match a central target for a duration of 500 ms, after which a new peripheral target appeared that replaced the starting target, accompanied by an auditory cue (660 Hz frequency, 250 ms long). This indicated the start of a movement period, lasting 2 s. A second auditory cue (880 Hz) signaled the start of a hold period of 1 s duration. Subjects were instructed to move and hold the cursor as close to the center of the target as possible. The time to act and match the target was kept deliberately short. While this constraint does not allow for an evaluation of maximum matching performance, it provides a measure of accuracy achieved within a limited time. This led to a more graded performance in single trials, thus allowing us to track the learning progress by means of the achieved accuracy. After the end of the hold period, a performance-related score was presented to the subjects (see section “Performance Measures”).

#### Experiment 1B

In the robotic hand control task, four finger flexion levels of the robotic hand were modulated: thumb, index finger, middle finger and ring/little finger; ring and little finger being coupled in their movement. Each one of these parameters was individually controlled by the normalized activation level $$ \check{y} $$ of one muscle and the four-dimensional posture vector **x** determined as $$ {\mathbf{x}} = {\check{\mathbf{y}}} $$, with $$ {\check{\mathbf{y}}} $$ being the vector of muscle activation levels $$ \left[ {\check{y}_{1} ,\check{y}_{2} ,\check{y}_{3}
,\check{y}_{4} } \right] $$. Relaxing all muscles opened the hand, which was used as a starting position. Targets in 2D space were replaced by target postures, appearing in the same pseudo-random order as the positional targets in part A. Similar to the cursor task, four target postures could be reached with activation of a single muscle, using 90% of comfortable contraction, whereas the other four required simultaneous activation of two muscles at accordant lower levels (≈64%). The analogy is illustrated in Fig. 2c by displaying target postures in the same circular arrangement as target positions in experiment 1A; the complete control space, however, was not two-dimensional but described by four independent parameters (i.e., finger flexions). Instead of two muscles working in opposite directions of one control dimension, as it was the case in experiment 1A, each control signal had an independent effect on the hand posture. Starting posture (open hand) and target postures were instructed by photographs of that posture on the laptop screen. The robotic hand was mounted about 40 cm behind the screen, so that subjects could comfortably observe both (Fig. 2a). New set-points for the robotic hand’s position controllers were provided at an average rate of 63 Hz, which well saturated the effective update capabilities of the prosthesis’ motors.

Holding the robotic hand at the starting posture for 500 ms initiated a trial. The same auditory cues as in the cursor control task were given at presentation of the target posture and beginning of the hold period. Subjects were instructed to match and hold the target posture with the robotic hand as closely as possible.

To illustrate experiment 1B, we provide two video clips, showing robotic hand control with Bayesian and linear filtering, respectively, along with control signals, as Supplementary Material.

### Experiment 2

A second experiment was designed to explore myoelectric control in the absence of visual feedback with application of the Bayesian and linear filtering methods. It consisted of a cursor control experiment, with the same layout as experiment 1A and was conducted with two groups of subjects: group B, utilizing the Bayesian estimator throughout most of the experiment, and group L, only experiencing the linear filter.

To confirm that both groups performed at similar levels, a short pre-learning block of 32 trials was set up, in which both groups used the linear filter. In the ensuing learning block of 320 trials, groups B and L used the Bayesian and linear filter, respectively. At the end of the experiment, another 64 trials were completed with both groups using the linear filter again (post-learning block). In learning and post-learning blocks, DoAs were identical to those in experiment 1A, whereas in the pre-learning block, a different set of non-intuitive DoAs was used to avoid experience being carried over into the learning block. If the choice of the filter had a persistent effect on behavioral strategy, groups should exhibit different performance in the post-learning block. Throughout the experiment, catch trials without visual feedback occurred with a chance of 25%. In catch trials, target positions were presented to the subjects, but they did neither receive visual feedback of cursor nor any other performance-related feedback. This ensured that only a feed-forward control, acquired during regular trials, could be applied, simulating blind control towards a known goal.

Targets were presented in a pseudo-random order with each target appearing once in eight trials, including two catch-trials without feedback; each target appeared in a catch trial once within 32 trials. The first four trials of both, pre-learning and learning block, were reserved for regular trials to allow subjects to experience the mapping of muscle activities before the first catch trial.

### Performance Measures

At the end of each trial, following the hold-period, in both experiments, with exception of catch trials in experiment 2, subjects were presented a score between 0 and 100, giving intuitive feedback of performance in the last trial. The score measure was designed to enable a direct comparison of accuracy of cursor and hand control. It was based on Euclidean distance of either the cursor position or the current hand posture to their respective targets. Due to mechanical constraints in the robotic hand, errors were much more limited in magnitude in part B of experiment 1 than in part A. To avoid a discrepancy between the two tasks for large errors, we imposed a limit on the Euclidean distance: Subjects at least had to reduce the distance to the target in order to achieve a positive score, whereas all larger errors resulted in a score of zero. Thus, the score was calculated as

3 $$ {\text{score}} = \left\{ \begin{array}{cc} 100 - [d({\mathbf{x,p}}_{{\mathbf{t}}} )/d({\mathbf{p}}_{{\mathbf{s}}} {\mathbf{,p}}_{{\mathbf{t}}} )] \times 100, & d\left( {{\mathbf{x,p}}_{{\mathbf{t}}} } \right) \le d\left({{\mathbf{p}}_{{\mathbf{s}}} {\mathbf{,p}}_{{\mathbf{t}}} }\right) \\ 0, & d\left( {\mathbf{x,p}}_{{\mathbf{t}}} \right) > d\left( {\mathbf{p}}_{{\mathbf{s}}} {\mathbf{,p}}_{{\mathbf{t}}} \right) \end{array}\right., $$

where *d*(**x**,**p**_**t**_) is the Euclidean distance between the two-dimensional cursor and target position (experiments 1A and 2) or between the current and target hand posture, as represented by vectors of four flexion levels (experiment 1B). The effector representation (cursor or hand posture) **x** was averaged over the time of the hold period. *d*(**p**_**s**_,**p**_**t**_) denotes the Euclidean distance between starting position **p**_**s**_ and target position or posture **p**_**t**_ by which *d*(**x**,**p**_**t**_) was normalized.

Scores relate to task performance. However, the effectors in experiments 1A and 2 had different degrees of freedom than in experiment 1B: cursor position was overdetermined by the four-dimensional control signal, whereas target postures corresponded to a unique pattern of four muscle contractions. To assess possible differences in control strategy, we therefore also compared tasks on the level of control signals. We evaluated the differences between the vector of normalized muscle activations ${\check{\mathbf{y}}}$ (cf. (1)), and an optimal signal ${\check{\mathbf{y}}}_{{{\mathbf{opt}}}}$ that represented the minimal muscle activation required to match the respective target position or posture **p**_**t**_:

4 $$ {\check{\mathbf{y}}}_{{{\mathbf{opt}}}}= \mathop {\arg \,\min}\limits_{{\left\{{\check{{\mathbf{y}}}|{\mathbf{x}}({\check{\mathbf{y}}})={\mathbf{p}}_{{\mathbf{t}}} } \right\}}} ||{\check{\mathbf{y}}} ||, $$

where ∥  ∥ specifies the Euclidean norm.

In the case of hand control only a single signal configuration could match a given target posture, whereas in cursor control ${\check{\mathbf{y} }}_{{{\mathbf{opt}}}}$ was given by a sparse activation of only those muscles that had DoAs either matching or neighboring (at 45°) target direction. We measured Euclidean distance between the pattern of average muscle activation ${\check{\mathbf{y} }}$ during the hold period and the optimal control signal ${\check{\mathbf{y} }}_{{{\mathbf{opt}}}}$. Normalized by the distance between the vector of no muscle contraction, corresponding to the starting position, ${\check{\mathbf{y} }}_{{{\mathbf{start}}}}$ and ${\check{\mathbf{y} }}_{{{\mathbf{opt}}}}$, the measure of signal optimality was calculated as:

5 $$ {\text{signal}}\;{\text{optimality}} = 1 - [d({\check{\mathbf{y} }},{\check{\mathbf{y} }}_{{{\mathbf{opt}}}}
)/d({\check{\mathbf{y} }}_{{{\mathbf{start}}}},{\check{\mathbf{y} }}_{{{\mathbf{opt}}}} )] $$

A value of 1 indicates optimal and accurate muscle activation; values below 0 indicate signal patterns that are farther from the optimal signal than resting levels. However, in the case of cursor control, low signal optimality does not necessarily correspond to a low performance since a redundant control space allows for different ways to match the target accurately.

### Subjects

In total, 21 right-handed subjects participated in this study. No subject took part in more than one experiment. They were free of any neurological or motor disorders and gave informed consent. The study was approved by the local ethics committee at Newcastle University.

Eight healthy volunteers (two female, six male) aged between 23 and 36 years (median: 28 years) participated in experiments 1A and 1B, which were run in close succession.

13 volunteers (10 female, three male) aged between 19 and 28 years (median: 23 years), participated in experiment 2.

### Statistical Analysis

Distributions of performance measures, to a large part, were non-Gaussian. We therefore characterized them by their median. Variability in such cases may be indicated by the upper and lower quartile. An approximation of a 95% confidence range of the median may be given as $\pm 1.96[(1.25 IQR)/(1.35 \sqrt N )],$ where *IQR* denotes the inter-quartile range and *N* the number of values in the test sample.^7^ In several cases we compared two groups of samples and tested for significant differences in their medians, using a Wilcoxon rank sum test for unpaired samples. For a family of *N* comparisons, the tests significance levels were adjusted by a factor of 1/*N* to yield a family-wise error rate ≤0.05 (Bonferroni correction).

## Results

### Experiment 1: Cursor vs. Hand Control

**Figure 3:**
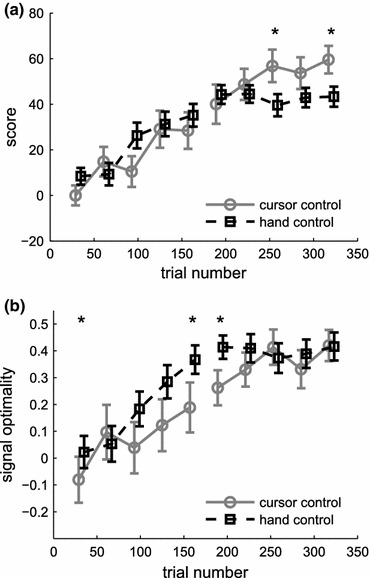
Learning curves of myoelectric control, experiment 1. (a) Median scores, representative of task performance, based on the Euclidean distance to target position (part A, cursor control, grey circles) or target posture (part B, hand control, black squares), over two consecutive blocks of 160 trials each. Medians were computed over periods of 32 consecutive trials and pooled from all subjects. (b) Learning curves measured by signal optimality (see Materials and Methods—“Performance Measures” section). Asterisks in both panels mark significant differences between the medians for cursor and hand control tasks within a set of 32 trials. Error bars indicate an estimate of the 95% confidence range of the median

We evaluated learning of the cursor and hand control tasks over time. Figure 3 shows average learning curves over two consecutive blocks of 160 trials each, for both parts of the experiment. Markers indicate median score over sets of consecutive 32 trials, pooling scores from all subjects. For each individual subject, the method of estimating muscle activation switched from the first to the second block of 160 trials. However, an equal number of subjects started with either of the two muscle activation methods.

While for both task conditions a steady improvement in task performance could be observed during the first block (Fig. 3a), myoelectric signals were closer to the optimal control in the hand control task than during cursor control (Fig. 3b). Scores of hand control only deviated significantly from cursor control at the end of the second block, where task performance peaked at a slightly lower level. However, at this time in the experiment, signal optimality was equally high in both tasks. Task performance also varied with the presented target; with targets that could be matched with the activity of just one muscle generally yielding higher scores (cf. Fig. S1, available as Supplementary Material).

**Figure 4:**
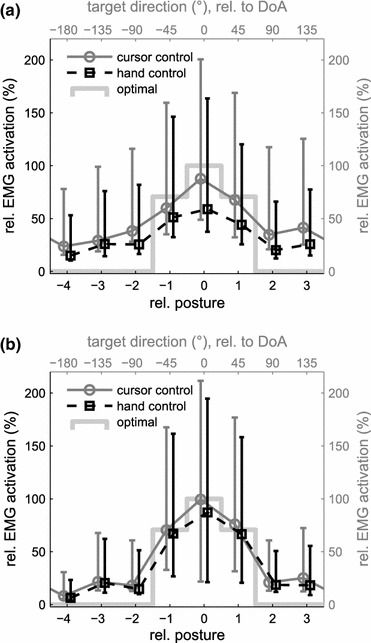
Changes in EMG tuning over time. (a) First 80 trials, (b) last 80 trials of a total 320 trials of experiment 1. Median EMG levels in the hold period are plotted over target directions (part A: cursor control, grey circles) or target posture (part B: hand control, black squares). The median was calculated over EMG levels from all muscles after target order was shifted so that DoA (cursor control) or the equivalent posture (hand control) corresponded to 0° or posture 0, respectively. Error bars display 25th and 75th percentiles. The light grey step function indicates the optimal pattern of minimal muscle activation (${\check{\mathbf{y} }}_{{{\mathbf{opt}}}}$)

Lower initial signal optimality in cursor control, without an equal loss in task relevant performance could have resulted from partial co-activation of muscles with opposite or perpendicular DoAs. For a confirmation we assessed muscle tuning functions that describe the relation between task goals and muscle activation. In our case it revealed how subjects’ behavior followed the relationship of myoelectric activity and the control output that was imposed by the experiment. Median muscle activation levels, as a percentage of the equivalent of target distance, are plotted across all targets in Fig. 4. Targets were aligned according to their direction for cursor control (experiment 1A) or an equivalent order of postures as shown in Fig. 2b for hand control (experiment 1B). Because tuning functions for single muscles would normally peak at different target directions or target postures, for a compound tuning of all muscles we shifted the alignments so that each muscle’s DoA (part A) or the equivalent posture (part B) corresponded to 0° or posture 0 on the horizontal axes of Fig. 4, respectively. During the early learning phase (Fig. 4a), tuning functions for cursor control (grey circles) were broad, had an elevated baseline and EMG levels showed high variability for targets far from the muscles’ DoA, whereas at the end of the experiment (Fig. 4b) median muscle tuning was close to the optimal activation pattern ${\check{\mathbf{y} }}_{{{\mathbf{opt}}}}$ (light grey line), with reduced variability in non-target directions, almost identical to the tuning function of hand control (black squares). In the early learning phase (Fig. 4a), however, variability of the control signals for robotic finger flexions, not needed to match the target posture (relative posture indices <−2 or >+2), was better contained during hand control than cursor control. This could be explained by the fact that deviations from the optimal signal were always apparent and detrimental to task performance in hand control.

While exploiting a redundant mapping in part A gave task performance a possible advantage over part B, the possibility to match cursor and target directly on the screen may have contributed to the higher peak performance in cursor control.

**Figure 5:**
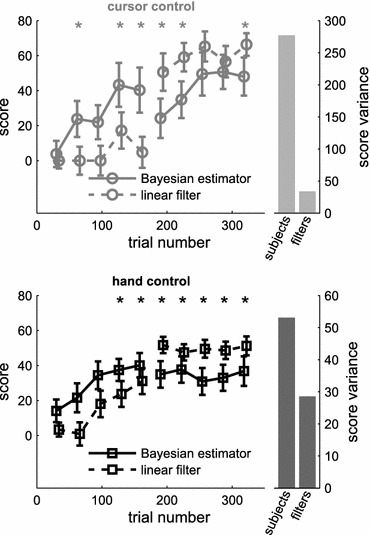
Comparison of filter conditions in experiment 1. Median scores over sets of 32 consecutive trials per subject (cf. Figure 3), split for the use of a Bayesian estimator (solid lines) and a linear filter (broken lines) in both cursor (top) and hand control task (bottom). Asterisks mark significant differences between blocks of different filter conditions (Wilcoxon rank sum test, family-wise error rate <0.05). Bars on the right of each panel compare the variance over subjects with the variance over filter conditions (average variance within sets of 32 trials per subject)

Learning rates, using either of two muscle activation estimators, were very similar (Fig. 5). However, it has to be noted that the experiment was not designed to compare absolute performance for the use of different filters. Task performance with different types of filters at a given time in the experiment could not be compared within the same subject because subjects switched filters between two consecutive blocks (first and last 160 trials). While we do find statistically significant differences in the medians within sets of 32 consecutive trials (asterisks in Fig. 5), this is more likely to reflect a difference over subjects. In fact, the variance over subjects largely exceeds the variance over filter types (right-hand panels in Fig. 5). A more adequate comparison of task performance with different muscle activation estimators is provided in experiment 2.

### Experiment 2: Control Without Visual Feedback

Experiment 2 intended to study how myoelectric control was maintained in the absence of visual feedback under use of either method to estimate muscle activation. Since learning of the task was comparable between cursor and hand control, we restricted experiment 2 to cursor control.

First, we compared scores, achieved in the first and last 48 trials with visual feedback of the learning block, to test whether participants of experiment 2 were able to acquire a significant amount of control. Four subjects who could not significantly improve their average score (*t*-test, *p* < 0.05) were identified as non-learners and excluded from further analysis since the ability to control the task was a prerequisite to make out differences in user control. Hence, we analysed data from five learning subjects in group L, who only used EMG processed with the linear filter and four in group B, who used the Bayesian filter to control cursor position in the learning block.

**Figure 6:**
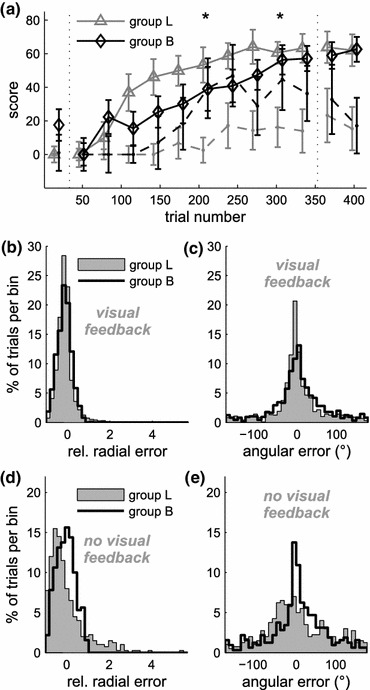
Performance in experiment 2. (a) Learning curves, displaying medians over trials of each condition within sets of 32 trials, pooled over group L (grey triangles), using the linear filter, and group B (black diamonds), using the Bayesian estimator. During the learning block (trials 33–352) subjects in group L and B used the linear and Bayesian estimators, respectively. Grey and black dots, connected by dashed lines correspond to trials with no visual feedback. Error bars denote standard error of the mean. Asterisks mark significant differences between trials of groups L and B in the no visual feedback condition. (b) Distributions of radial errors in the condition with visual feedback during the learning block (group L: grey area; group B: black outline). Errors are based on average cursor positions in target direction during the hold period. Negative values indicate undershooting, positive values overshooting the target. (e) Distributions of angular deviations in trials with visual feedback. (d) Distributions of radial errors in the condition without visual feedback. (e) Distribution of angular error without visual feedback

Figure 6a gives an overview of task performance of the two groups. We tested for differences in the median performance between groups L and B in sets of 32 consecutive trials, containing 24 trials with visual feedback and eight—one to each target—without. Absence of any significant differences in the pre-learning block confirmed that both groups had comparable average skill. Similar performance in the post-learning block, with and without visual feedback, indicates that the choice of filters in the learning block had no lasting effect on feedback performance and feed-forward control. However, in trials without visual feedback in the second half of the learning block, scores for users of the Bayesian estimator (group B) were significantly higher (two sets, marked by asterisks in Fig. 6a) than for those using the linear filter. When visual feedback was available, subject performance in the learning block was without significant differences between both groups.

In a closer look on cursor deviations from the target, we calculated radial errors as the average deviation from the target during the hold phase, on an axis in target direction (Fig. 6b). Distributions of radial errors are almost identical between both groups, as long as visual feedback is available. The same is true for errors in direction (angular errors, Fig. 6c). When no visual feedback is provided, however, the distribution of radial errors (Fig. 6d) from group L is skewed towards negative values, demonstrating a tendency to undershoot the target, whereas in some trials also large overshoots were observed. For group B on the other hand, radial errors were still almost symmetrically distributed around zero. Slight differences were also present in the distributions of angular errors without visual feedback (Fig. 6e). However, their circular standard deviations were not significantly different between groups. Therefore, the lowered accuracy of subjects using the linear filter (group L) without vision, seen in Fig. 6a can mostly be attributed to larger deviations in radial direction, including both overshoot and undershoot.

**Figure 7:**
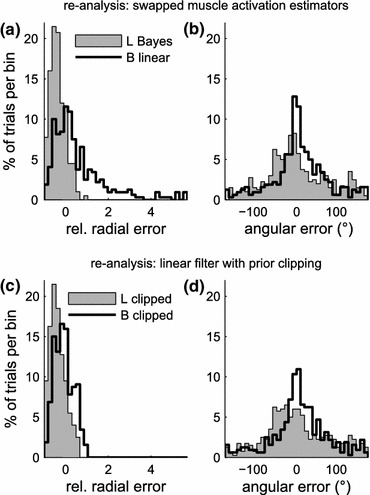
Learning-block trials without visual feedback; offline re-analysis with different EMG filters. (a, b) Distribution of radial and angular errors with Bayesian estimator and linear filter exchanged; group L re-analysed with the Bayesian estimator, group B re-analysed with the linear filter. (c, d) Distributions of radial and angular errors, both groups (L and B) re-analysed with a linear filter and prior clipping of the raw EMG (±3*σ*)

During trials without visual feedback, behavior cannot be affected by the type of EMG processing, as subjects are unaware of its consequences. However, it might have led to the adaptation of a different control strategy during previous trials with feedback. To distinguish between differences in groups L and B that were caused by a passive influence of filter structure and those caused by a behavioral strategy, we re-analysed trials without visual feedback with linear filters exchanged for Bayesian estimators and *vice versa* (Figs. 7a and 7b). The overshoot, present in trials without visual feedback (Fig. 7a) in group L was eliminated by a switch to a Bayesian estimator in the post hoc analysis, which in turn increased the percentage of undershooting trials even further. A switch to a linear filter for group B reproduced the overshoot that was previously observed with the original filter setting for group L (see Fig. 6d). The tendency of group L to undershoot in a large number of trials, on the other hand, was not reproduced. The distribution of angular errors was not affected by a post hoc change in EMG processing (Fig. 7b). Thus, the lack of large overshoots (group B) can be assumed to be a property of the Bayesian filtering process itself, whereas group L’s tendency to undershoot in a majority of trials reflects a strategy that was acquired while using the linear filter, possibly as a strategy to counterbalance occasional large overshoots.

One specific feature of the Bayesian estimator that could possibly explain a limit to the overshoot is the fact that EMG signals were clipped at ±3 standard deviations, which originally served as a measure to facilitate modeling of the EMG’s density function. We tested this assumption by re-analyzing the EMG of groups L and B, using a linear filter in both cases, but with previous clipping of the EMG, in the same way it was done for Bayesian filtering. In fact, this measure eliminated the large overshoots from the original linear filter. The distribution of radial errors for both groups (Fig. 7c) closely matched the cases when the Bayesian estimator was used (cf. Fig. 7a for group L; Fig. 6d for group B). This indicates that reduced overshoot can be attributed to the initial clipping procedure.

## Discussion

We present a translation of a myoelectric cursor control paradigm to direct proportional control of a dexterous hand prosthesis. The four-muscle control scheme of the two-dimensional cursor task could be well translated into real-time control of a robotic hand: we observed similar dynamics of learning the control, and very similar initial levels of accuracy for the cursor control task (experiment 1A) and the robotic hand control task (experiment 1B).

This implies that control mechanisms, relevant to the use of prosthetic hands, can be studied in the well-established and easily implementable framework of center-out cursor movements. An example for such an application is the study of feed-forward control in experiment 2, which revealed that even without visual feedback, the proportional control scheme is still applicable. In light of experiment 1, we are confident that this would also apply to subjects, training to control a robotic hand. Ultimately, however, this claim still needs validation through further experiments with myoelectric control of prosthetic hands that include trials without visual feedback.

We believe a myoelectric-controlled cursor task could further be a valuable and inexpensive tool to identify the most promising set of muscles, prior to the fitting a prosthetic hand. In this line, virtual reality tasks have already been suggested to train patients in the control of myoelectric prostheses using a classification approach^18^ and are also available in commercial systems, such as MyoBoy (Otto Bock, Duderstadt, Germany) or Biosim (Touch Bionics, Livingston, UK). However, the direct and continuous feedback, offered by a proportional controller may lend itself better to biofeedback training.

In the absence of direct feedback, transient high amplitudes in the EMG can lead to larger than intended control signals, when processing with a simple linear filter. Clipping high values in the EMG prior to filtering, efficiently avoids this problem. We expect that subjects, training with a linear filter with prior clipping, would also develop a more balanced feed-forward control in the same way another group of subjects did, using the Bayesian estimator.

In summary, we believe that our approach can be a valuable tool for the study of myoelectric prostheses.

### Implications for Myoelectric Prosthetics

A one-to-one mapping of muscles to actuators of a robotic hand, as demonstrated in our study, may not be viable for prosthetic hands with many degrees of freedom, if only a few muscles are available for stable recordings. Further, a large number of controlling muscles may increase necessary training time and cognitive effort. Hence, for actual prosthetic applications, a more promising path of action might be to try and reduce the dimensionality in the prosthesis’ control space for common hand movements.^12^ In spite of the reduced complexity, this can still allow the access of a continuum of relevant hand postures: Matrone *et* *al.* 5,6 used principal component analysis of 50 different grasps of a prosthetic hand to identify two components that were sufficient to grasp a large variety of objects. This approach might further be able to constrain hand configurations so that unfavorable postures e.g., paths of different fingers crossing each other, will be avoided.

We deliberately avoided intuitive DoAs in our experiments to slow down the learning process and better observe improvements over time. In real-life prosthetic applications the choice of recordable muscle signals may be severely restricted due to the nature of the patients’ disabilities, precluding intuitive control. These cases are, to some degree, better emulated by a non-intuitive design, such as ours. While a more intuitive control is easier to learn^13^ and is therefore preferable, efficient proportional control is not ruled out by limitations in recording sites. A biomimetic approach, relying on pattern recognition of grasps may be much more limited in the use of the available muscles.^4^

We used a set of four intrinsic hand muscles to obtain four well separable recordings of EMG. This, on first glance, prohibits a direct transfer of this concept to transradial amputees where hand muscles are missing. However, today, a great percentage of patients only require a partial hand prosthesis with active digits, in which case some intrinsic hand muscles may still be intact and could be used for myoelectric control. In fact, i-limb digits (Touch Bionics) already provide this possibility. Moreover, novel techniques for chronically implantable myoelectric electrodes such as IMES^19^ or epimysial electrode arrays^14^ could allow for direct access to several remaining forearm muscles and even surpass surface recordings of intrinsic hand muscles in terms of stability and independence of individual muscle signals.

Kuiken and colleagues^2^,^3^,^8^ explored a surgical technique, known as targeted muscle reinnervation, where remaining distal motor nerves are redirected to proximal muscles after amputation. This permitted multiple recordings of surface EMG from a confined area, through which subjects could learn to control several degrees of freedom of a prosthetic device. As the control of the distal motor system is more flexible to learn new neuromotor mappings,^9^,^13^,^17^ targeted muscle reinnervation by distal motor nerves seems particularly promising to adopt a control scheme, similar to the one presented in this study, for cases of trans-humeral or trans-radial amputation.

## Electronic supplementary material

Below is the link to the electronic supplementary material.

Supplementary material (MP4 10689 kb)

Supplementary material (MP4 10193 kb)

Supplementary material (TXT 2 kb)

Supplementary material (PDF 200 kb)

Supplementary material (PDF 235 kb)

## Abbreviations

APB: Abductor pollicis brevis muscle
1DI: First dorsal interosseous muscle
3DI: Third dorsal interosseus muscle
ADM: Abductor digiti minimi muscle
DoA: Direction of action
