# Supplementary material for: Abstract and Proportional Myoelectric Control for Multi-Fingered Hand Prostheses
Source: Ann Biomed Eng. 2013 Aug 9;41(12):2687–98. doi: 10.1007/s10439-013-0876-5 (PMC3825263; doi:10.1007/s10439-013-0876-5)
Supplement: Supplementary file 4 — Supplementary material (PDF 200 kb) [file 10439_2013_876_MOESM4_ESM.pdf]

Supplementary Figures

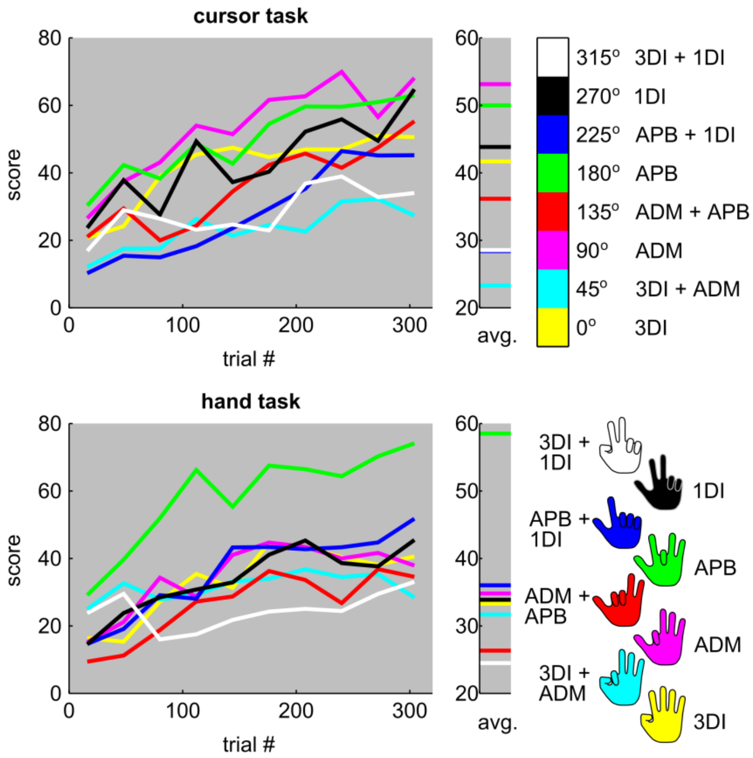

FIGURE S1. Differential performance for different targets. Scores of experiment 1A (top) and 1B (bottom), averaged over trials to a specific target (coded in colour, see legend on the right), within sets of 32 consecutive trials (four per target), from all subjects. Narrow panels to the right of the main panel display the mean score over all trials. Legend assigns target directions (top) or target postures (bottom) to colours and shows which muscles had to be contracted to reach the respective target. Targets that required the activation of two muscles (in white, blue, red and cyan) generally received lower scores. Some differences between cursor task (exp. 1A) and hand task (exp. 1B) in the order of target preference suggest that different target-muscle associations may have been perceived as intuitive.
