# Supplementary material for: Abstract and Proportional Myoelectric Control for Multi-Fingered Hand Prostheses
Source: Ann Biomed Eng. 2013 Aug 9;41(12):2687–98. doi: 10.1007/s10439-013-0876-5 (PMC3825263; doi:10.1007/s10439-013-0876-5)
Supplement: Supplementary file 5 — Supplementary material (PDF 235 kb) [file 10439_2013_876_MOESM5_ESM.pdf]

Supplementary Methods

Linear Filter

The linear filter used in this study conforms to a causal moving average filter of rectified EMG measurements, where the filtered signal $y(t)$ is given by:

$$y(t) = \int_{t-w}^{t} |EMG(t-\tau)| \mathrm{d}\tau \tag{S1}$$

where t represents time and w is the width of the filter (in this study: 750 ms).

In discrete time, with a filter width of n samples, y at time step k is given by:

$$y_k = \frac{1}{n} \sum_{i=k-n+1}^{k} |EMG_i| \tag{S2}$$

Bayesian estimator

The following is a brief summary of the Bayesian filter algorithm described by Sanger[13].

Rectified EMG measurements, in the presence of a driving signal z, are modelled by a random process with an exponential probability density:

$$emg = |EMG| \tag{S3}$$

$$\mathrm{P}(emg|z) = \frac{e^{-emg/z}}{z} \tag{S4}$$

The driving signal z is modelled as a combination of a diffusion process W with diffusion rate α and a counting process N_β with rate constant β:

$$\mathrm{d}z = \alpha(\mathrm{dW}) + (U - z)\mathrm{dN}_\beta \tag{S5}$$

U is a random variable, evenly distributed in [0, 1]; z is limited to the same interval [0, 1].

An approximate solution for the evolution of the density of z is given by

$$\frac{\partial \mathrm{p}(z,t)}{\partial t} = \alpha \frac{\partial^2 \mathrm{p}(z,t)}{\partial z^2} + \beta[1 - \mathrm{p}(z,t)] \tag{S6}$$

where $\frac{\partial^2 \mathrm{p}(z,t)}{\partial z^2}$ is the density evolution for a diffusion process and β indicates the probability of a jump to an arbitrary value of z.

Recursive algorithm in discrete time and z being discretized into bins of width ε:

1. Initialize the prior $\mathrm{p}(z,0) = 1$
2. Forward propagate $\mathrm{p}(z,t-) \approx \alpha\mathrm{p}(z-\varepsilon,t-1) + (1-2\alpha)\mathrm{p}(z,t-1) + \alpha\mathrm{p}(z+\varepsilon,t-1) + \beta + (1-\beta)\mathrm{p}(z,t-1)$

3. Measure the rectified EMG signal and clip values exceeding a maximum value m
4. Calculate the posterior likelihood function $\mathrm{P}(z,t) \approx \mathrm{P}(emg|z)\mathrm{p}(z,t-)$, where $\mathrm{P}(emg|z)$ is given by the exponential model in (4)
5. Output the signal estimate $y[z(t)] = \text{argmax } \mathrm{P}(z,t)$
6. Divide $\mathrm{p}(z,t)$ by a constant C, so that $\int \mathrm{p}(z,t)\mathrm{d}z = 1$
7. Repeat from step 2
